# A novel role of Krüppel-like factor 4 in Zhikong scallop *Chlamys farreri* during spermatogenesis

**DOI:** 10.1371/journal.pone.0180351

**Published:** 2017-06-30

**Authors:** Dandan Yang, Zhifeng Zhang, Shaoshuai Liang, Qiankun Yang, Yingrui Wang, Zhenkui Qin

**Affiliations:** 1 Key Laboratory of Marine Genetics and Breeding (Ocean University of China), Ministry of Education, Qingdao, China; 2 Laboratory for Marine Fisheries Science and Food Production Processes, Qingdao National Laboratory for Marine Science and Technology, Qingdao, China; Baylor College of Medicine, UNITED STATES

## Abstract

Krüppel-like factor 4 (KLF4) is a kind of zinc finger transcription factor, which is involved in terminal differentiation of epithelial cells and reprogramming of somatic cells to induced pluripotent stem (iPS) cells in mammals. In the present study, we identified a full-length cDNA of *Klf4* in Zhikong scallop *Chamys farreri* (*Cf-Klf4*) and found that *Cf-Klf4* presented a sexual dimorphic expression characteristic in *C*. *farreri* gonads. *Cf-Klf4* expression was significantly higher in testes than in ovaries from growing stage to mature stage detected by quantitative real-time PCR, and was located in male gametes, except for spermatozoa during spermatogenesis through *in situ* hybridization and immunohistochemistry, while no positive signal was visible in female gametes during oogenesis. Furthermore, the knockdown of *Cf-Klf4* in testes by means of *in vivo* RNA interference led to an obviously developmental retardance, lower gonadosomatic index, less male gametes and more apoptotic spermatocytes. Interestingly, we found that two out of eight scallops showed a hermaphroditic phenotype characteristic of male-to-female sex reversal when the *Klf4* mRNA and protein levels were knocked down in males. These results verified that *Klf4* plays an important role in testis functional maintenance and is necessary in spermatogenesis of *C*. *farreri*.

## Introduction

Krüppel-like factor 4 (KLF4), a gut-enriched Krüppel-like factor (GKLF) or epithelial zinc finger (EZF), was firstly identified from the mouse serum-deprivation fibroblast cell cDNA library [1]. It has the highly conversed domains of KLF family, the DNA-binding domains with three classical Cys_2_/His_2_ zinc fingers at the C-terminus binding GC-box or CACCC-box of the target DNA [2, 3], and two Krüppel-links, TGEKP(Y/F) X which connect zinc fingers [4]. The distinctions between KLF4 and other KLF members are some functional domains, including transcriptional activation domain, transcriptional repression domain, nuclear localization signal (NLS), and a potential PEST domain in mammals [5, 6]. These specific functional domains enable KLF4 to work as transcription regulators. Segre, et al. [7] and Katz, et al. [8] reported that losing of KLF4 leads to the deficiency of skin barrier and the reduction of goblet cells, indicating its indispensability in epidermal differentiation. During tumorigenesis, KLF4 works as a crucial transcription factor playing distinctive roles, such as in gastrointestinal and lung cancers, as a tumor suppressor, it regulates the expression of cell cycle-related genes [9], induces cell apoptosis [6] or inhibits the activity of telomerase [10]; or as an oncogene, it suppresses the *cdh1/skp2/p27* pathway to promote cell proliferation in breast cancer [11, 12]. Moreover, KLF4 is also an important reprogramme factor which can induce some somatic cells to generate the induced pluripotent stem (iPS) cells [13, 14].

Up to now, studies about *Klf4* related to sexual development were only reported in mouse and human. Researchers found that *Klf4* is strongly expressed in post-meiotic germ cells of mouse and human testes, indicating its role in testicular differentiation in mammals [15–18]. Godmann, et al. [18] found that although lacking of *Klf4* in germ cells of mouse testis generated by the *Cre-loxP* system does not impair spermiogenesis, some genes related in differentiation, proliferation and cell cycle etc. exhibited up- and down-regulated expressions in the mutants, implying its potential function in spermatogenesis.

Bivalve mollusks possess fascinatingly diverse modes of reproduction, including dioecism (e.g., *Chlamys farreri*), hermaphrodite (e.g., *Argopecten irradias*) and even sex reversal (*Crassostrea gigas*), suggesting that bivalves are good animal models for studying sex determination and reproductive regulation [19]. In previous research, some sex-related genes, such as *dmrt1*, *dmrt2*, *dmrt4*, *SoxH*, *foxl2*, *β-catenin*, have been identified in some bivalves, and present sexual dimorphic expression characteristics; while *17β-HSD8* presents similar expression between males and females [19–25]. However, the understanding of particular key genes involving in sex is still very limited. In the present study, we identified a full-length cDNA of *Klf4* in the Zhikong scallop *C*. *farreri* (*Cf-Klf4*), which is a dioecism with stable sex composition and important commercial shellfish in Northern China, and revealed that *Cf-Klf4* was specifically expressed in germ cells of *C*. *farreri* testes. Furthermore, we determined that *Cf-Klf4* participated in the regulation of spermatogenesis and maintenance of testis function in *C*. *farreri* tested by means of RNA interference (RNAi). Our data provide important clues for better understanding of the molecular mechanism about gametogenesis and sex formation in shellfish.

## Materials and methods

### Ethics statement

The collection and handling of the scallops were performed in accordance with the Ocean University of China Institutional Animal Care and Use Committee (OUC-IACUC) and the local government. No specific permissions were required for the described studies, and the studies did not involve endangered or protected species.

### Animals and sampling

Healthy male and female scallops *C*. *farreri* with mean shell height 6.39±0.41 cm were collected from Shazikou Bay (Qingdao, China). Gonads were dissected and weighed for subsequent analysis. Parts of the gonads were immediately frozen in liquid nitrogen and stored at -80°C. The remainders were fixed in 4% paraformaldehyde in 0.01 M phosphate buffered saline (PBS) at 4°C for 20 h, then dehydrated with serial methanol (25%, 50%, 75% and 100%) diluted in 0.01 M PBS and stored in 100% methanol at −20°C.

According to the morphologic characteristics described by Liu, et al. [26] and Liao, et al. [27], the gonads were grouped into four stages based on the histological structure and the gonadosomatic index (GSI = gonad weight/soft tissue body weight×100), the resting stage (GSI = 3.57±0.80 for ovary and 3.73±0.25 for testis), the proliferative stage (GSI = 3.98±0.98 for ovary and 4.07±0.72 for testis), the growing stage (GSI = 6.87±0.62 for ovary and 6.90±0.58 for testis) and the mature stage (GSI = 9.62±1.47 for ovary and 9.76±1.46 for testis).

### Histology

Samples were dehydrated in an ascending gradient of ethanol, cleared in xylene and embedded in paraffin wax. Sections (5 μm thick) were fixed to a microscope slide with 0.1% polylysine at 37°C for 10 h. The procedure of histology was followed by the description of Liu, et al. [26]. The sections were observed and photographed using a Nikon E80i microscope (Nikon, Tokyo, Japan).

### RNA extraction

Total RNA was extracted from gonads of the four stages using Trizol RNA extraction kit (Invitrogen, CA, USA) according to the manufacturer’s protocol. After removal of contaminant DNA, with DNase I (Takara, Otsu, Japan), purified RNA was quality-checked and quantified with gel electrophoresis and spectrophotometry.

### Cloning of target cDNA in *C*. *farreri*

The 3’- and 5’- RACE ready first-strand cDNA was synthesized from 1 μg total RNA of adult testis at proliferative stage using SMARTer^TM^ RACE kit (Clontech, CA, USA) according to the manufacturer’s instruction. The specific primers, GSP-F (5’-GTATGCTGGCGTCCTCAGTGAACAGAGC-3’) and GSP-R (5’- GTGTACAGCTACTGGAGTTGTCTGCTGG-3’), were designed according to a 2,301 bp cDNA fragment which was retrieved from *C*. *farreri* transcriptome (SRX218546). Nested PCR was conducted using 50× diluted primary PCR product as the template and the nested primers GSNP-F (5’- GTGTACAGCTACTGGAGTTGTCTGCTGG -3’) and GSNP-R (5’- TGAACGCAAGTCAGACAGTCTTC-3’) to clone the 5’ and 3’ regions of *Klf4* cDNA, respectively. The PCR products were sequenced and then assembled with SeqMan Pro (DNA STAR, WI, USA).

### Sequence analysis

The sequence similarity of KLF4 with those from other species was analyzed using BLAST program at NCBI (http://blast.ncbi.nlm.nih.gov/Blast.cgi). Multiple alignments were analyzed using the CLUSTAL X2 software. A phylogenetic tree was constructed using MEGA 6.0.6 with 1000 bootstrap trials.

### Quantitative real-time PCR (qRT-PCR)

qRT-PCR analysis was employed to determine the expression level of *Klf4* mRNA in testes and ovaries at different stages. Two specific primers, qPCR-F 5’- GAAAGCGACAGACAAGCCAC-3’ and qPCR-R 5’- GGTAAGTTCATCAGAGCGAGCA-3’ were designed based on the full-length sequence of *Klf4* for amplifying a 164 bp gene-specific product, and elongation factor 1 alpha (*ef-1α*) of *C*. *farreri* was used as a reference gene [28]. The amplification was carried out in a total volume of 20 μl using Roche LightCycler 480 Real-Time PCR System (Roche, Basel, Switzerland) and SYBR Green Master Mix (Takara, Dalian, China) following the manufacturer’s instruction. 2^-ΔΔCt^ method was used to analyze the relative expression levels of *Cf-Klf4* mRNA. All data were presented as mean ± SEM from five samples with three parallel repetitions, and all qRT-PCR assays were validated in compliance with “the MIQE guidelines” [29].

### *In situ* hybridization (ISH)

A cDNA fragment of 564 bp was amplified with two specific primers, ISH-F 5’-GGCGAATTCGATCACTATGATGTCGTA-3’ and ISH-R 5’-CCGAAGCTTGATGAATTGATGTCAGGA-3’, and DIG-labeled RNA sense and antisense probes of *Cf-Klf4* were generated using a DIG RNA labeling Kit (SP6/T7) (Roche, Basel, Switzerland) following the manufacturer’s instruction. DIG-labeled RNA probes of *C*. *farreri foxl2* (*Cf-foxl2*) were synthesized as described in Liu, et al. [22]. Sections of the gonadal tissues were made following the method in “Histology”. *In situ* hybridization was performed as described [30], with the modifications that samples were digested for 10 min at 37°C with 2 μg/ml protease K and counterstained with 1% neutral red. The sections were observed and photographed using a Nikon E80i microscope (Nikon, Tokyo, Japan).

### Immunohistochemistry

The open reading frame of *Cf-Klf4* cDNA was amplified with the sense primer 5‘-GAGCTCATGGATAACGGTTCGTTG -3’ (SacI site underlined) and the antisense primer 5‘-CTCGAGTATGTGGCGTTTCATATG-3’ (XhoI site underlined). The prokaryotic expression and purification of *C*. *farreri* KLF4 were performed following protocols as described [25]. The polyclonal antibody of *C*. *farreri* KLF4 against New Zealand white rabbits was produced by Sangon Biotech (Shanghai, China). The serum antibody titer was determined by indirect enzyme-linked immunoassay, and the antisera were aliquoted and stored at −80°C.

Total protein was extracted from approximately 100 mg of *C*. *farreri* testis at mature stage using total protein extraction kit (CW Biotech, Beijing, China) according to manufacturer’s instruction. Western blot was performed in triplicate for detecting specificity of the *Cf-*KLF4 antibody and the level of *Cf*-KLF4 protein as described by Ma, et al. [31]. The polyclonal antibody of Rabbit anti-β-Actin (CW Biotech, Beijing, China) was used as an internal control to calibrate the total extractive proteins. The *Cf-*KLF4 antibody (anti-KLF4) was diluted as 1:1000 by PBS/Tween-20 plus 5% skimmed milk powder.

Sections of the gonadal tissues were made following the method in “Histology”. Immunohistochemistry was conducted following protocols described by Ma, et al. [31], with the modification that sections were incubated in PBS/Tween-20 plus 5% skimmed milk powder for 1 h. The control group was performed using negative serum, which was extracted before injecting purified recombinant *Cf-*KLF4 into New Zealand white rabbits, instead of the anti-KLF4. Observation and digital images were taken with a Nikon E80i microscope (Nikon, Tokyo, Japan).

### RNAi assay

#### dsRNA synthesis

The procedure was performed as described by Suzuki, et al. [32] with some modifications. Two primers, RNAi-F 5’-TAATACGACTCACTATAGGGAGACGTGAGCCTCTTGGTGTT-3’ (T7 promoter underlined) and RNAi-R 5’-TAATACGACTCACTATAGGGAGTGCATGAAATGATGCGTAG-3’ (T7 promoter underlined), were designed according to *Cf-Klf4* cDNA sequence to amplify a specific fragment (522 bp). PCR products were gel-purified and then transcribed *in vitro* using the T7 MEGAscript RNAi Kit (Ambion, Austin, USA) to synthesis double-stranded RNA (dsRNA). The dsRNAs were phenol/chloroform-extracted, ethanol-precipitated, and suspended in RNase-free PBS (pH 7.4). Purified RNA was quality-checked and quantified with gel electrophoresis and spectrophotometry.

#### dsRNA administration and sampling

Healthy male scallops *C*. *farreri* with mean shell height 6.25±0.32 cm were purchased from Shazikou Bay (Qingdao, China). Seventy-five male scallops at the proliferative stage were evenly assigned and maintained in three aquaria with 430 L filtered, aerated seawater at 16.1±0.4°C, respectively. Unicellular algae *Platymonas helgolandica* and *Chaetoceros muelleri* were fed daily and water was renewed twice a day during the experiment. For each group with twenty-five scallops, 80 μl PBS containing 50 μg *Klf4* dsRNA (dsKLF4 group) or 80 μl PBS without dsRNA (PBS group) were injected into the adductor muscle at T0 (initiation of this assay) and T7 (the 7^th^ day), respectively. The PBS and blank (no injection) groups were used as control, respectively. The testes were dissected and weighted at 3 d (five scallops for each group), 10 d (five scallops for each group) and 24 d (eight scallops for each group), respectively. The procedure of sampling was the same as “Animals and sampling”.

#### Determining the number and composition of germ cells in the follicles of gonads

Gonadal sections from five scallops of each group were made following the method in “Histology”. To inspect the number and composition of germ cells in gonads after RNAi, all the germ cells in the follicles was counted by randomly observing five sights (2500 μm^2^ for each) in histological sections of gonads and percentage of germ cells at different developmental stages was calculated.

#### TdT-mediated dUTP Nick-End Labeling (TUNEL)

The TUNEL assay was conducted to determine cell apoptosis in the testes after RNAi using a DeadEnd^TM^ Colorimetric TUNEL System Kit (Promega, Madison, USA) following the manufacturer’s instruction. Sections of the testis tissues were made following the method in “Histology”. Observation and digital images were taken with a Nikon E80i microscope (Nikon Co., Tokyo, Japan).

### Statistical analysis

All data were presented as means ± SEM. Significant differences between means were tested using one-way analysis of variance (ANOVA) followed by Tukey’s HSD test (SPSS software version 18.0; SPSS Inc., Chicago, USA), and the significant level was set at *P*<0.05 in all cases.

## Results

### Sequence analysis of *C*. *farreri Klf4*

**Fig 1:**
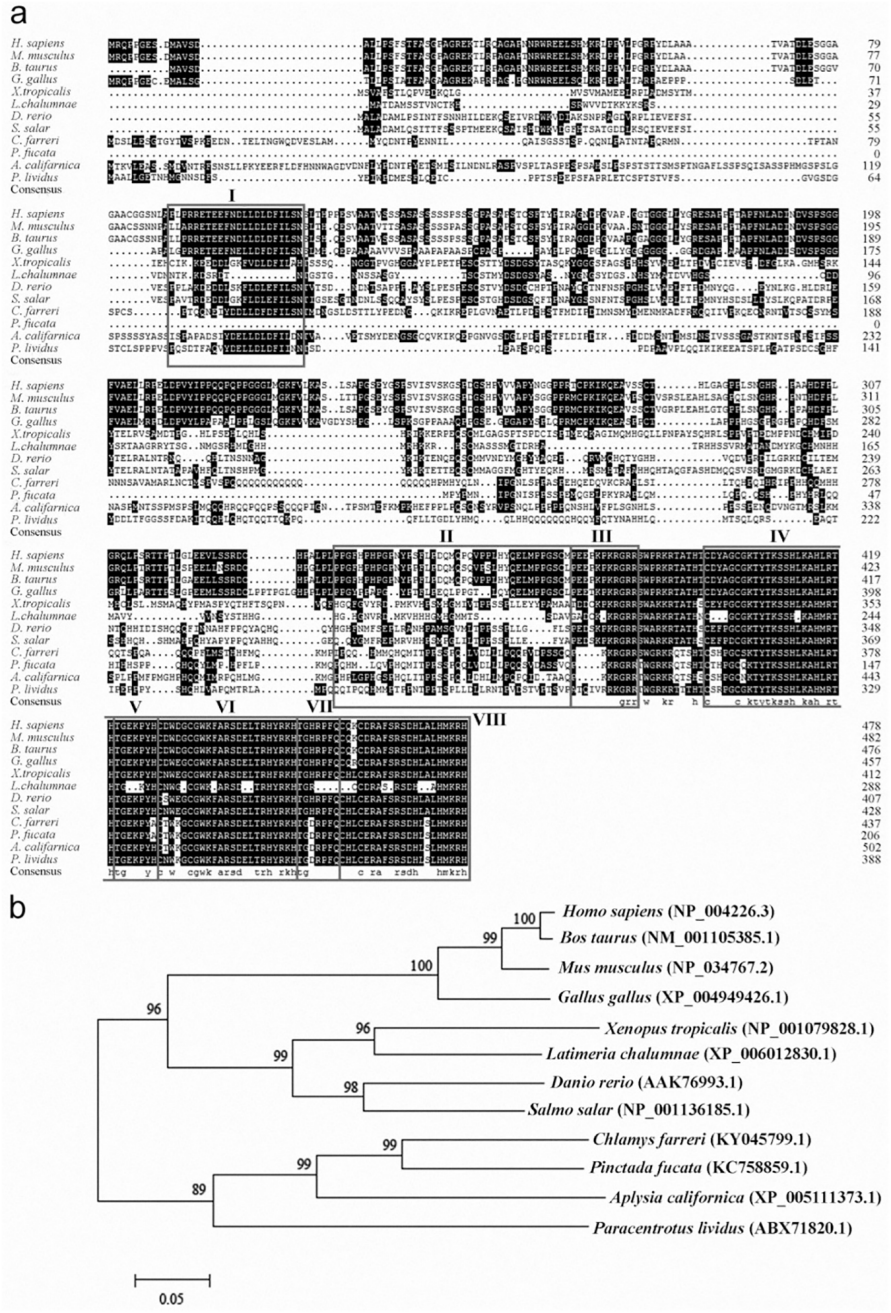
**Multiple sequence alignment (a) and phylogenetic analysis (b) among the KLF4 sequences from different species.** Identical residues are highlighted in black. The transcriptional activation domain, transcriptional repression domain and nuclear localization signal are in box I to III. The three Cys_2_His_2_ type zinc fingers are in box IV, VI and VIII, its linkers are in box V and VII.

Two fragments of 1,168 bp and 1,563 bp were cloned from 5’- and 3’- RACE, respectively. The full-length cDNA sequence was 2,610 bp (GenBank accession number: KY045799.1), containing a 1,314-bp open reading frame (ORF) encoding a putative protein of 437 amino acid residues, a 96-bp 5’-untranslated region (UTR) and a 1,202-bp 3’-UTR. Molecular mass of the putative protein was 50.1 kDa and the isoelectric point was 7.68. The deduced amino acid sequence contained three conversed DNA-binding domains and two linkers of KLF family, as well as three putative functional domains of KLF4 (Fig 1A). Furthermore, the deduced amino acid sequence was homologous with other known KLF4s, which was 75% identical to *Pinctada fucata*, 63% to *Paracentrotus lividus* and *Latimeria chalumnae*, and 71% to *Salmo salar*. Especially, KLF4s from different organisms are highly conserved in their three classical Cys_2_/His_2_ zinc fingers and nuclear localization signals by a ClustalX2 alignment (Fig 1A).

The phylogenic tree demonstrated that *C*. *farreri* KLF4 clustered primarily with that of mollusca *P*. *fucata*, *Aplysia californica* and *P*. *lividus*, successively, and then formed a subcluster with the branch formed by vertebrates, including *Homo sapiens*, *Mus musculus*, *Gallus gallus*, *Xenopus tropicalis*, *D*. *rerio* and so on. (Fig 1B).

### Temporal expression of *Klf4* in gonads at different stages

**Fig 2:**
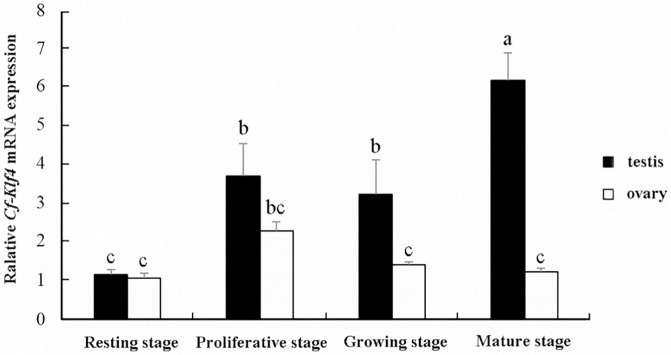
Expression of *Cf-Klf4* mRNA in *C*. *farreri* gonads at different stages detected by qRT-PCR. Data are indicated as the mean ± SEM from five independent samples with triplicate. The expression level in the testes at resting stage is set as 1.00 to calibrate the relative levels in gonads at other stages. Different letters indicate significant differences (*P* < 0.05).

qRT-PCR results showed the levels of *C*. *farreri Klf4* mRNA in testes grew significantly (*P*<0.05) from resting stage to proliferative stage, with a 3.5-fold increasement, and then it kept a relative stable level from the proliferative stage to growing stage and reached the maximum in mature stage, which was 6-fold higher than that in resting stage (*P<*0.05). However, no significant difference of *Cf-Klf4* expression level in ovaries was presented during oogenesis (Fig 2, S1 Table). Moreover except for the resting stage, the *Cf-Klf4* mRNA levels were significantly different (*P*<0.05) between testes and ovaries, which was 1.5-fold, 3-fold and 6-fold higher in the testes than that in the ovaries at the proliferative, growing and mature stage, respectively (Fig 2).

### Cyto-location of *Cf-Klf4* mRNA and protein during gametogenesis

**Fig 3:**
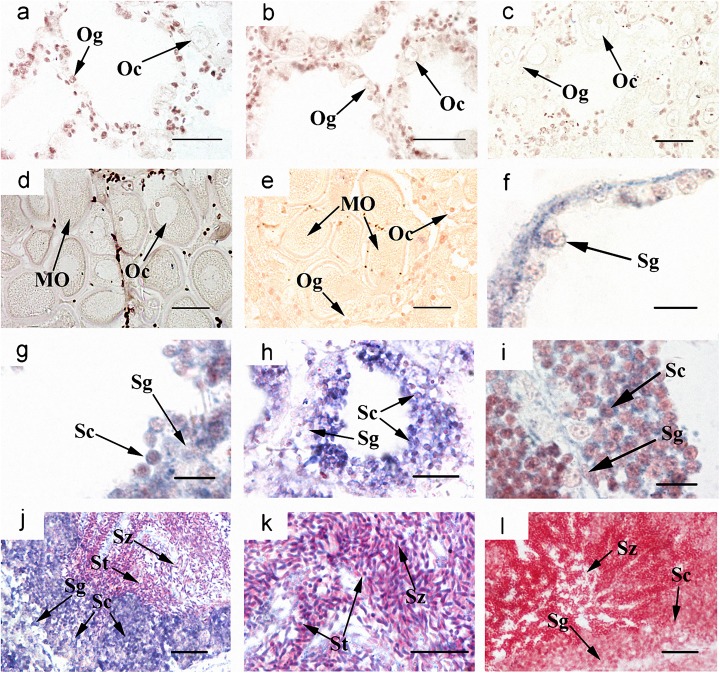
Localization of *Cf-Klf4* mRNA in gonads of *C*. *farreri* demonstrated by *in situ* hybridization. Positive signals with an antisense probe are indicated in *blue*; controls with sense probe are indicated in mature ovary (e) and testis (l). (a)-(e), ovary; (a), resting stage; (b), proliferative stage; (c), growing stage; (d), mature stage. (f)-(l), testis; (f), resting stage; (g), proliferative stage; (h), growing stage; (j), mature stage; (i) and (k), magnified image of (h) and (j), respectively. Og, oogonium; Oc, oocyte; MO, mature oocyte; Sg, spermatogonium; Sc, spermatocyte; St, spermatid; Sz, spermatozoon. Bar, 40 μm for a-e; 20 μm for (h), (j), (l); 10 μm for others.

**Fig 4:**
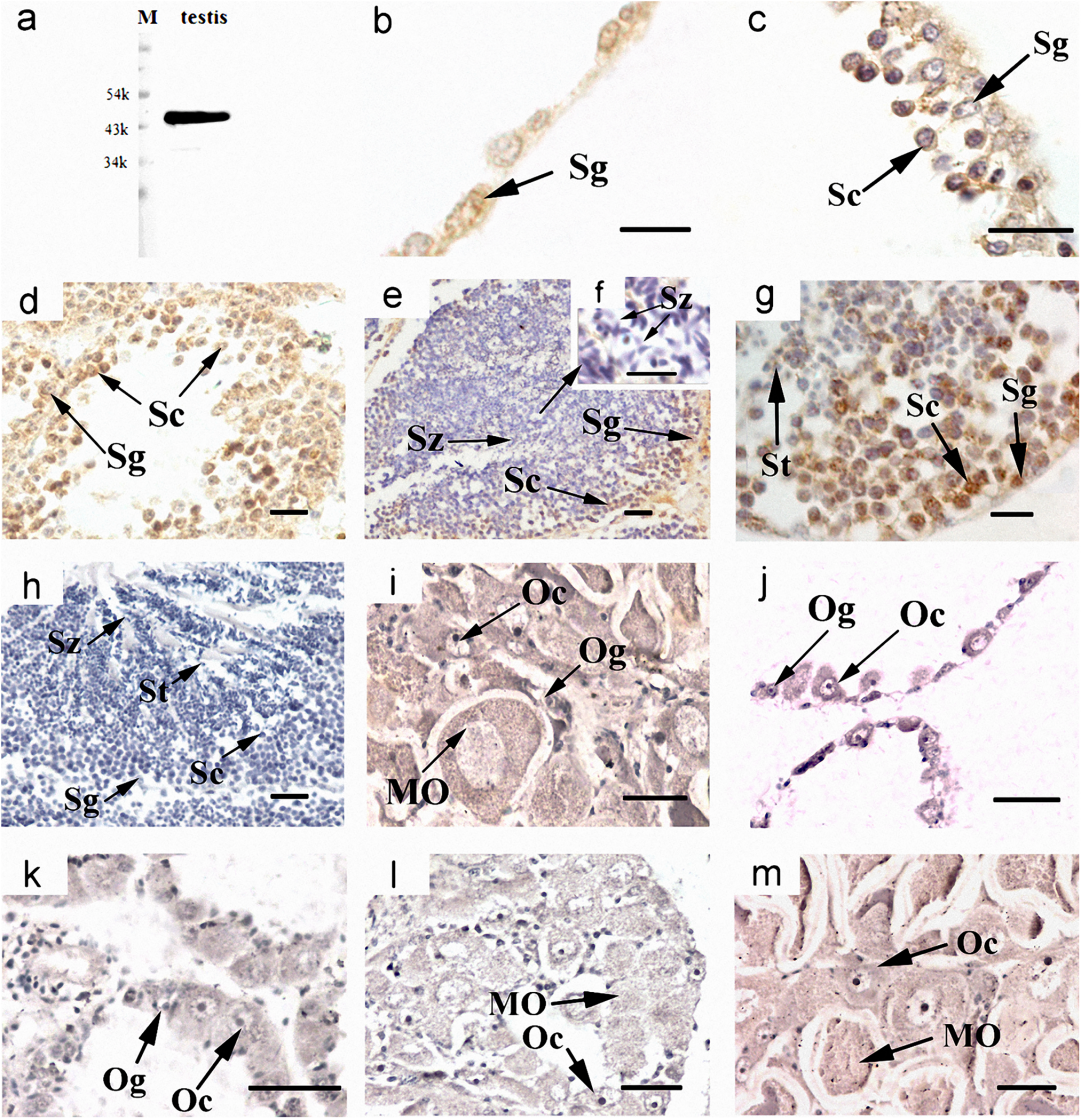
Localization of *Cf-*KLF4 protein in gonads of *C*. *farreri* demonstrated by immunohistochemistry. Positive signals with anti-KLF4 are indicated in *brown*; controls with negative serum are indicated in mature testis (h) and ovary (i). (a), specific test of KLF4 polyclonal antibody shows that only a specific band appears, meaning KLF4 polyclonal antibody is specific. (b)-(h), testis; (b), resting stage; (c), proliferative stage; (d), growing stage; (e), mature stage; (f), (g), magnified image of (e). (i)-(m), ovary; (j), resting stage; (k), proliferative stage; (l), growing stage; (m), mature stage; Og, oogonium; Oc, oocyte; MO, mature oocyte; Sg, spermatogonium; Sc, spermatocyte; St, spermatid; Sz, spermatozoon. Bar, 10 μm for (b), (f); 20 μm for others.

The cyto-location of *C*. *farreri Klf4* in the testes was different from that in the ovaries during gametogenesis (Figs 3 and 4). In testes, *Cf-Klf4* transcripts were visible in all germ cells (Fig 3F–3K), and a similar location of *Cf-*KLF4 protein was also presented except in spermatozoa (Fig 4B–4G). However, the positive signal in oogonia and oocytes was hardly visible or faint at either mRNA or protein level (Figs 3A–3D and 4J–4M).

### *Klf4* knockdown led to a testis developmental retardance in *C*. *farreri*

**Fig 5:**
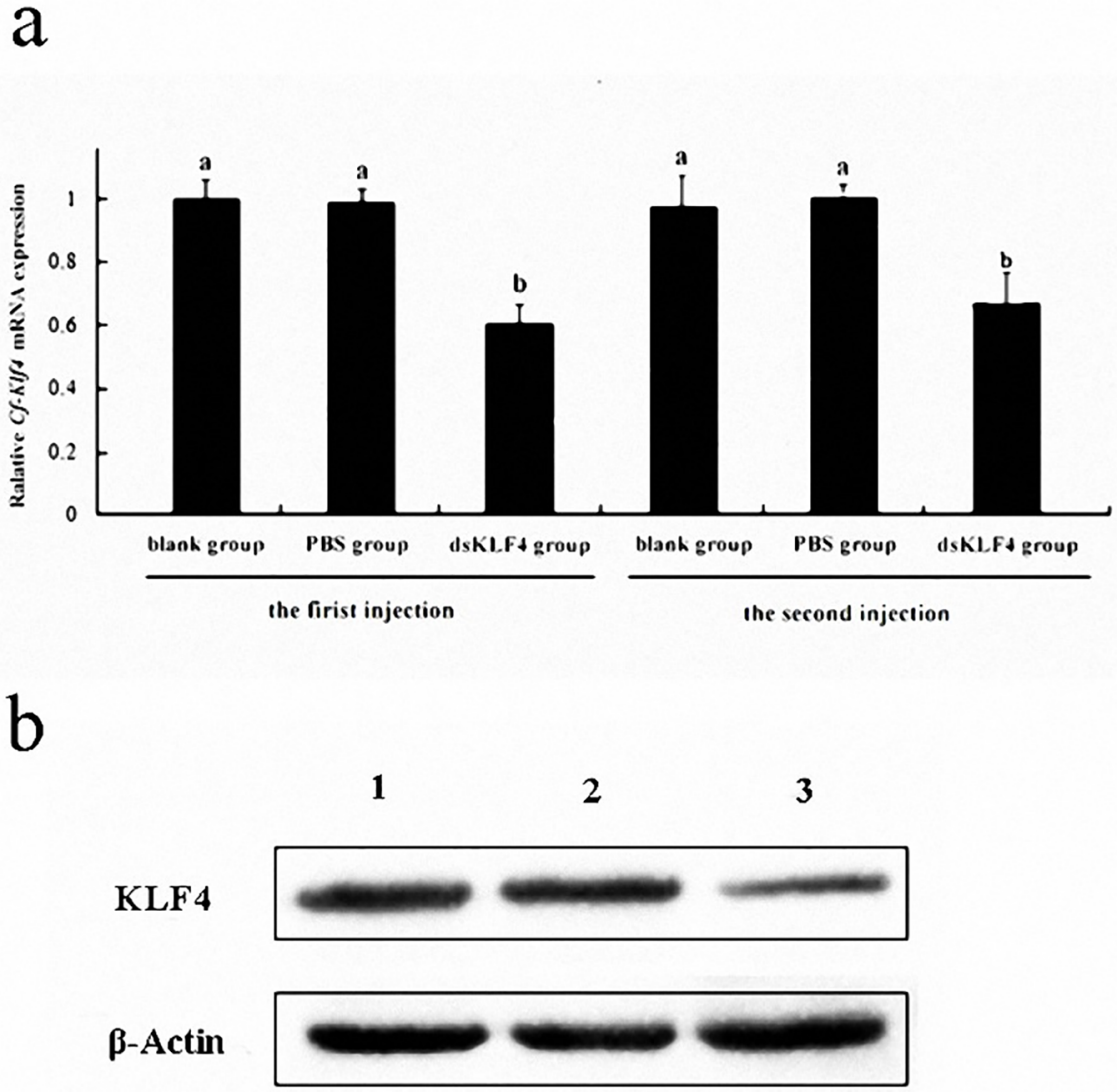
**Level of the *Cf-Klf4* mRNA and *Cf*-KLF4 protein in *C*. *farreri* testes for 72 h post-injection detected by qRT-PCR (a) and Western blot (b).** (a) Level of the *Cf-Klf4* mRNA detected by qRT-PCR. Data are indicated as the mean ± SEM from five independent samples with triplicate. The expression level in the testes of blank group is set as 1.00 to calibrate the relative levels in testes of other groups. Different letters indicate significant differences (*P* < 0.05). (b) Level of the *Cf*-KLF4 protein for 72 h post the second injection detected by Western blot. 1, blank group; 2, PBS group; 3, dsKLF4 group.

qRT-PCR detected that the *Cf-Klf4* transcript level in the testes of dsKLF4 group was significantly decreased (*P*<0.05), which was only 60% of controls, the PBS or blank group (Fig 5A, S2 Table). Meanwhile, the level of *Cf*-KLF4 protein in dsKLF4 group was decreased when compared to the PBS and blank groups detected by Western blot (Fig 5B). No significant difference was presented between two control groups (Fig 5).

**Fig 6:**
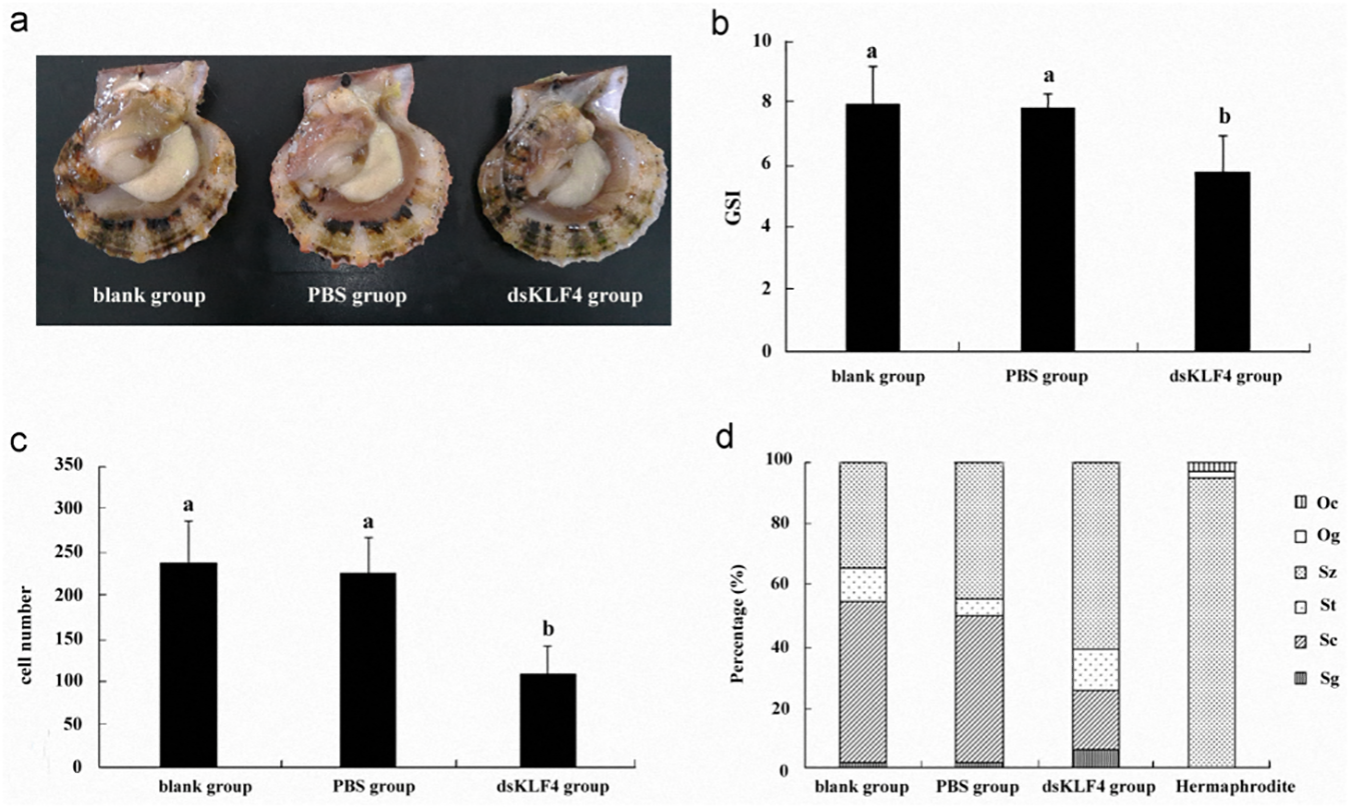
Characteristics of *C*. *farreri* testes when *Cf-Klf4* knockdown for 24 d. (a), phenotype; (b), GSI (*n* = 8); (c), Gamete number in testis follicles of a 2500 μm^2^ area (*n* = 5, five sights each individual, respectively); (d), percentage of various types of germ cells in a total number of 200 gametes (*n* = 5, five sights each individual, respectively). Data are means ± SEM. Different letters indicate significant differences (*P* < 0.05).

We found the phenotype of scallops in the dsKLF4 group presented obvious differences from the PBS and blank groups when *Cf-Klf4* mRNA in the male scallops was knockdown for 24 d. In the dsKLF4 scallops, the testes were translucent and wizened, and the size was smaller than that of the control groups (Fig 6A). The GSI of the dsKLF4 scallops (5.77±1.16) was significantly (*P*<0.05) lower than that of the PBS (7.86±0.44) or the blank scallops (7.97±1.20) (Fig 6B, S3A Table).

**Fig 7:**
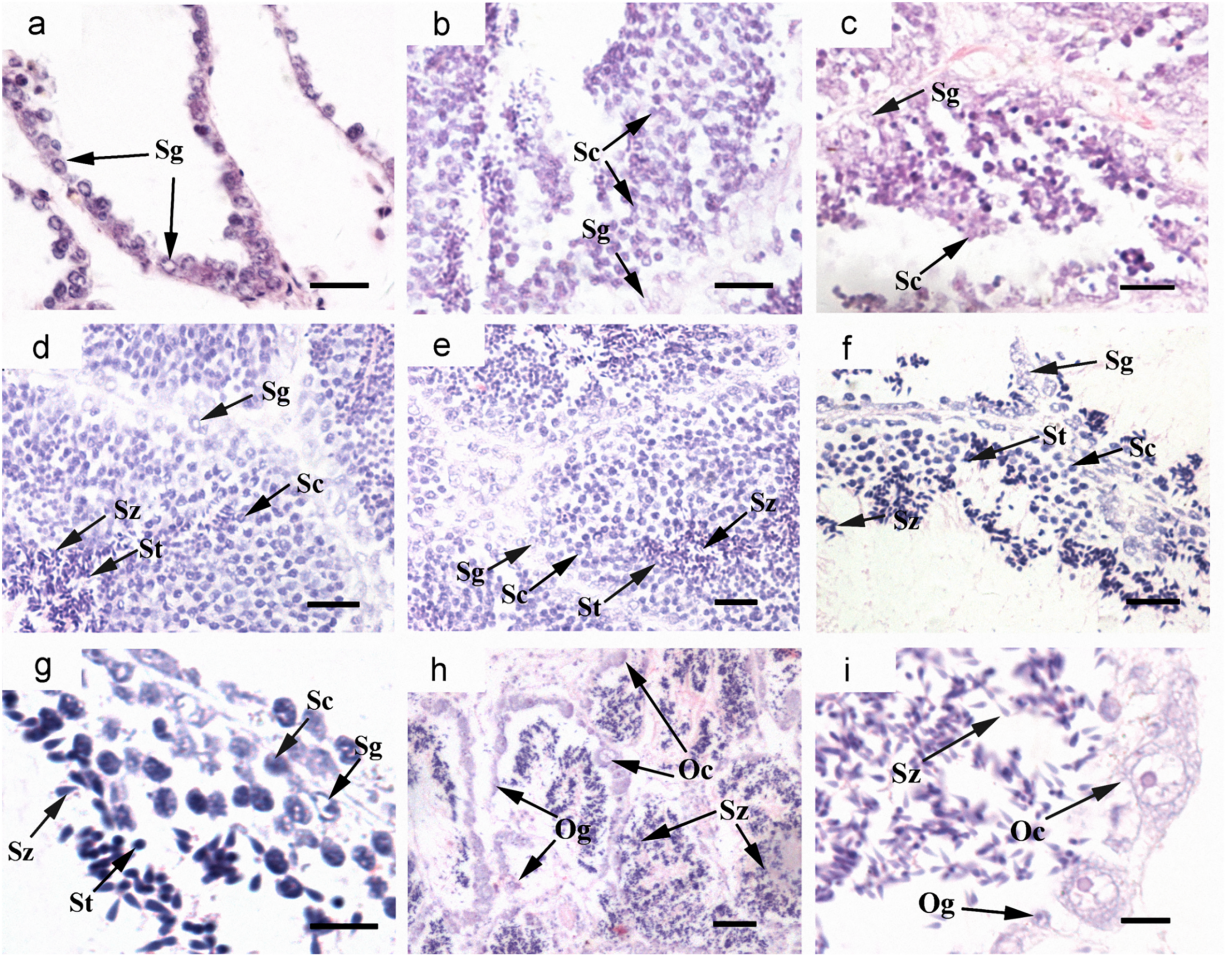
Histological characteristics of *C*. *farreri* testes in RNAi experiment. (a), testes at the start of experiment; (b), testes at the 10^th^ day of blank group; (c), testes at the 10^th^ day of dsKLF4 group; (d)-(i), testes after RNAi for 24 days. (d), blank group; (e), PBS group; (f), dsKLF4 group; (h), the hermaphroditic gonads in the dsKLF4 group after RNAi for 24 days; (g) and (i), magnified images of (f) and (h), respectively. Sg, spermatogonium; Sc, spermatocyte; St, spermatid; Sz, spermatozoon; Og, oogonium; Oc, oocyte. Bar, 10 μm for (g) and (i); 20 μm for others.

Histological examination showed the characteristics of the testis follicles in the dsKLF4 group were obviously different from the control groups at day 24. In the blank and PBS groups, all testes developed to mature stage, and the follicles were filled with spermatogenic cells of different developmental stages, meanwhile spermatozoa were arranged radially with tails pointing towards the center of the follicle (Fig 7D–7E). In the dsKLF4 testes, however, the follicular cavity was obviously empty compared to that of the controls, and only a few male gametes were in the follicle wall (Fig 7F). The quantity of the germ cells in the follicles of dsKLF4 testes was significantly less than that of the control groups (*P*<0.05). In the 2500 μm^2^ view of testis sections under microscope, the cell number in the dsKLF4 group was 108.2±32.2, accounting for only 46.8% of the blank group (Fig 6C, S3B Table), while there were no significant differences between PBS group (225.0±41.4) and blank group (237.7±48.2). In addition, the germ cell composition in dsKLF4 testes was obviously different from controls, which was 6%, 19% and 60% of spermatogonia, spermatocytes and spermatozoa, respectively, in dsKLF4 group, compared to 1.6%, ~50% and ~45% of that in PBS and blank groups (Fig 6D, S3C Table).

**Fig 8:**
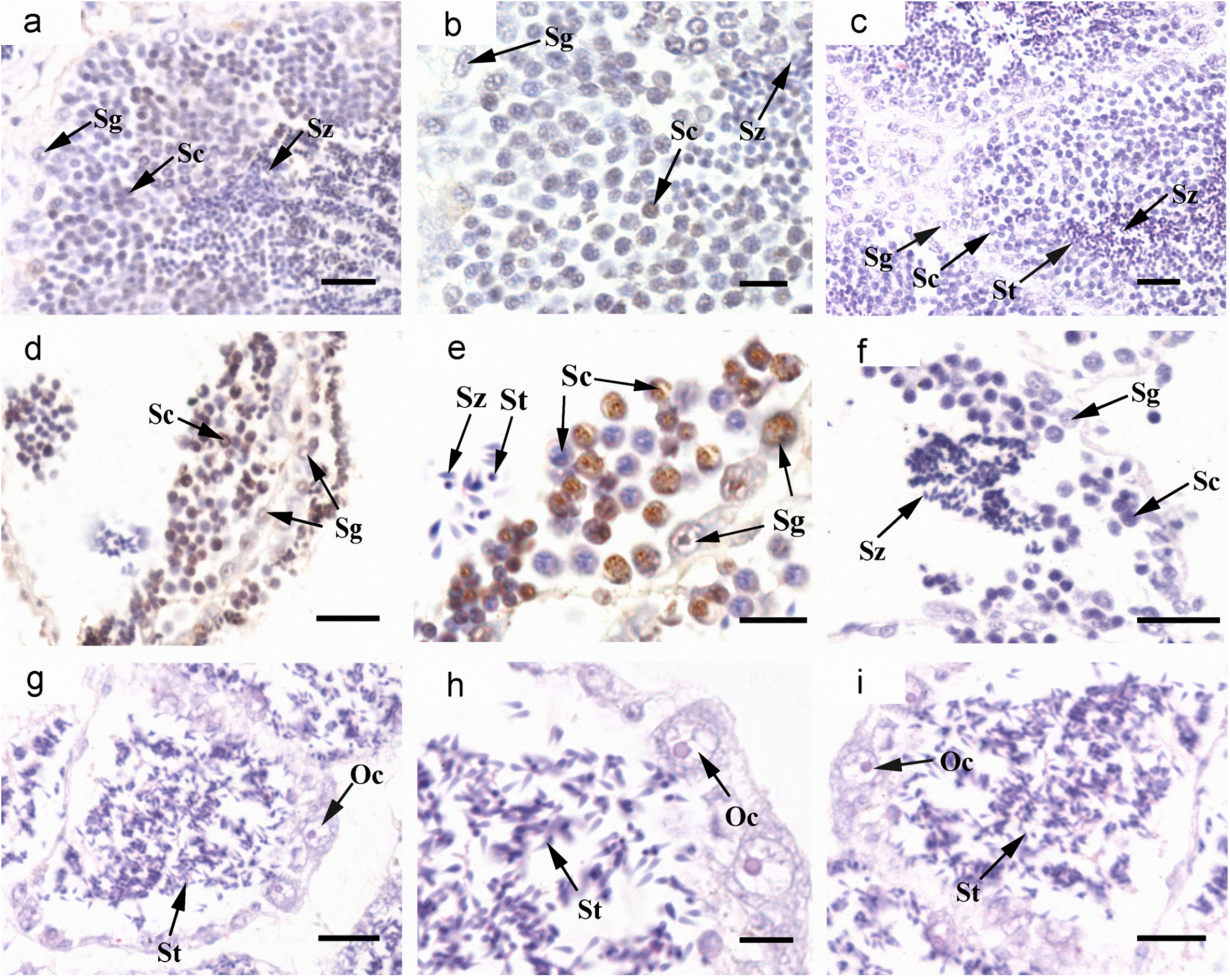
Cell apoptosis in the testes demonstrated by TUNEL after RNAi for 24 days. Positive signals are indicated in *brown*. (a)-(c), blank group; (d)-(f), dsKLF4 group; (g)-(i), the hermaphroditic gonad in the dsKLF4 group; (b), (e), (h), magnified images of the gonads; (c), (f), (i), negative control without terminal deoxynucleotidyl transferase of TUNEL experiment. Sg, spermatogonium; Sc, spermatocyte; St, spermatid; Sz, spermatozoon; Og, oogonium; Oc, oocyte. Bar, 10 μm for (b), (e), (h); 20 μm for others.

TUNEL detection revealed that most spermatocytes and a few spermatogonia in dsKLF4 testes presented obvious positive signals of apoptosis (Fig 8D), while no obvious signals were visible in spermatids and spermatozoa. In the blank group testes, only several apoptosis cells were detected (Fig 8A).

Interestingly, we observed two of the eight scallops in the dsKLF4 group presented unique gonadal structure appearing to be hermaphroditic at day 24. In each follicle of testes in these two scallops, the majority of germ cells in the follicular cavity was spermatozoa (approximate 95%, Fig 6D), moreover, oogonium-like and oocyte-like cells instead of spermatogonia and spermatocytes scattered in the follicle wall (Fig 7H). No TUNEL positive signal was detected in these testes (Fig 8G).

**Fig 9:**
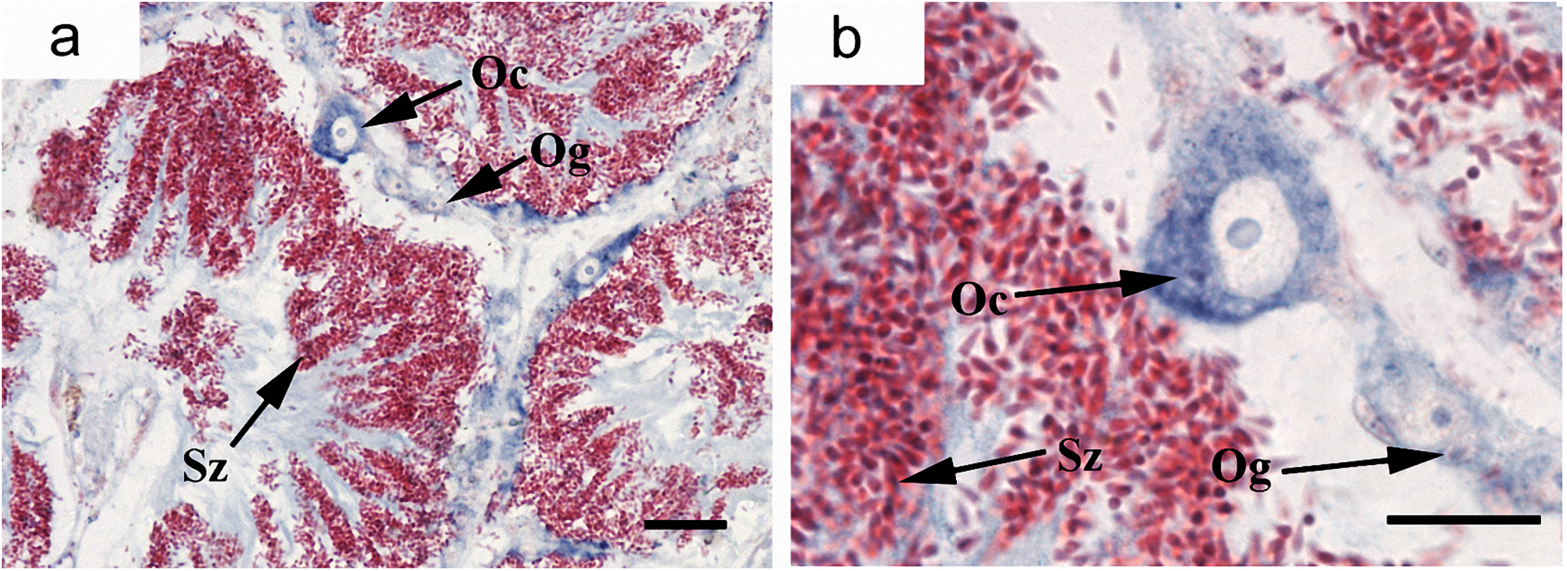
Location of a female-specific gene *Cf-foxl2* demonstrated by *in situ* hybridization in the hermaphroditic gonads of dsKLF4 group. Positive signals with an antisense probe are indicated in *blue*. (b), the magnified image of (a). Sz, spermatozoon; Og, oogonium; Oc, oocyte. Bar, 20 μm.

We used a *C*. *farreri* female-specific gene *foxl2* [22] to further identify the types of oogonium-like and oocyte-like cells in the hermaphroditic-like gonad by means of *in situ* hybridization (Fig 9). Results exhibited that female gametes specific signals were also presented in these cells, which suggested these two scallops were hermaphroditic.

## Discussion

In this study, we firstly reported the characteristics of *Cf-Klf4* sexual dimorphic expression in *C*. *farreri* testes and ovaries, which is different from mammals. Furthermore, the *Klf4* knock-down in *C*. *farreri* testes led to the retardance of spermatogenesis, even male-to-female sex reversal, indicating its essential roles in spermatogenesis.

### *Cf-Klf4* presents a characteristic of sexually dimorphic expression in *C*. *farreri* during gametogenesis

Up to now, expression of *Klf4* in testis and ovary was only studied in human and mouse, which indicated a sexually dimorphic characteristic. In human, the *Klf4* expression level is about 2-fold higher in testes than ovaries [16]. In mouse, levels of *Klf4* mRNA and protein are also high in testes [15, 33]. Moreover, KLF4 has been reported only in testis, and presented only in post-meiotic germ cells of mouse and human adult testes or postnatal mouse testes [15, 16, 18]. In this study, we presented a sexually dimorphic expression of *Klf4* in *C*. *farreri* testis and ovary. However, it is obviously different in expression level and cellular localization between the mammals and the scallop. In *C*. *farreri*, the level of *Cf-Klf4* mRNA in testes was 6–fold higher than that of ovaries at mature stage, and *Cf-Klf4* was located in all the male germ cells and hardly visible in *C*. *farreri* ovaries (Figs 3A–3E and 4I–4M). Therefore, we hypothesized *Klf4* might also involve in spermatogenesis and its role might be different between the mammals and the shellfish.

### *Cf-Klf4* regulates early spermatogenesis in *C*. *farreri*

Godmann, et al. [18] reported that deletion of *Klf4* in germ cells does not impair spermiogenesis in the mouse based on histological structure, GSI, fertility and testosterone level in the testes compared with that of the control, although some genes involving in differentiation, proliferation and cell cycle are up- or down-regulated. In the present study, testis development and spermatogenesis were obviously retardanted when *Cf-Klf4* mRNA and *Cf-*KLF4 protein were knocked down in the testes of *C*. *farreri*, which were demonstrated with the significantly decreased GSI (*P*<0.05) (Fig 6B), the empty follicle cavity (Fig 7F), and significantly reduced number of spermatogenic cells in the dsKLF4 testes (*P*<0.05) (Fig 6C). Therefore, we determined that *Cf-Klf4* participates the regulation of spermatogenesis and its role is different between the scallop *C*. *farreri* and the mouse *M*. *musculus*.

Shields, et al. [1] reported that KLF4 is an important differentiation factor of epithelia and involved in cell cycle regulation. Chen, et al. [34] revealed that induced KLF4 expression is associated with up-regulation of many genes involving in the cell cycle arrest and down-regulation of genes in promoting cell proliferation. Moreover, it has been well-known that during spermatogenesis, spermatogonia differentiate into primary spermatocytes, which subsequently generate the secondary spermatocytes through meiosis I and spermatids through meiosis II. Therefore, proportion of the spermatocytes in follicles is relatively stable in mature testes. Nevertheless, we found in the present study that the proportion of spermatocytes was obviously reduced in the dsKLF4 testes comparing to the blank group (Fig 7C and 7F), and the extent of the reduction gradually increased with the experiment proceeded (Fig 6D). Meanwhile, the proportion of spermatogonia increased in the dsKLF4 group (Fig 6D). Obviously, the knockdown of *Cf-Klf4* mRNA broke the dynamic equilibrium among the spermatogenic cells. Furthermore, almost half of spermatocytes and a few spermatogonia were in apoptosis status, while little positive signals were found in control groups by means of TUNEL detection (Fig 8A and 8B). In view of the above mentioned facts, we suggested that the *Cf-Klf4* regulates early spermatogenesis, and might promote the differentiation of post-mitosis spermatogonia and post-meiotic spermatocytes as well as the apoptosis of spermatocytes.

### *Klf4* maintains the testis function

The sex determination and formation is a vital and essential progress during individual development, with several key genes or a serial of genes involved. Up to now, only several genetic models for sex determination and formation has been proposed in model organisms, such as *M*. *musculus*, *Drosophila melanogaster* and *Caenorhabditis elegans* [35–37]. In bivalve mollusks, studies on sex determination and formation are still in its infancy, even the relevant key genes have not yet been identified. Laurent, et al. [38] demonstrated that function loss of *foxl2*, a female functional maintenance gene is sufficient to cause an XX female-to-male sex reversal in the goat. *Dmy*, a *dmrt1* homologue in the medaka *Oryzias latipe* has been identified to be male sex-determining gene based on its mutation can cause a male-to-female sex reversal [39]. In the present study, two hermaphroditic scallops were observed in the dsKLF4 gonads in which the oogonia and oocytes presenting *foxl2* positive signals (Fig 9) scattered along the walls in all the follicles, as well as spermatozoa distributed in the follicular cavity (Fig 7G). Liao, et al. [27] and our years of research demonstrated that the Zhikong scallop *C*. *farreri* is a kind of dioecious bivalve and its gender composition is stable. Moreover, Wu, et al. [40] reported when the clam *Paphia undulata* occurs sex reversal from male-to-female, male germ cells are in the center of the follicle while female germ cells are scattered in the follicle wall. While, when it is female-to-male sex reversal, female germ cells are in the center of the follicle while male germ cells are scattered in the follicle wall. Hereby in the present study, we confirmed the hermaphroditic gonads in the dsKLF4 group is male-to-female sex reversal, and suggested that *Kfl4* is essential for the spermatogenesis and the maintenance of male gonadal function in *C*. *farreri*.

## Conclusions

In the present work, we identified a *Klf4* full-length cDNA of 2,610 bp in *C*. *farreri*. The *Cf-Klf4* expression presents a sexually dimorphic characteristic during gametogenesis, and is specifically visible in spermatogonia, spermatocytes and spermatids of *C*. *farreri* testes, which is different from that in human beings and mice. Furthermore, the *Cf-Klf4* should participate in the regulation of early spermatogenesis and play an important role in maintaining the testis function based on the *Cf-Klf4* RNAi analysis. In the future, it remains to study molecular mechanism of *Klf4* regulating spermatogenesis in shellfish.

## Supporting information

S1 Table Expression of *Cf-Klf4* mRNA in *C*. *farreri* gonads at different stages detected by qRT-PCR. (XLSX)

S2 Table Level of the *Cf-Klf4* mRNA in *C*. *farreri* testes for 72 h post-injection detected by qRT-PCR. (XLSX)

S3 Table Characteristics of *C*. *farreri* testes when *Cf-Klf4* knockdown for 24 d. (a), GSI; (b), Gamete number in testis follicles of a 2500 μm^2^ area; (c), percentage of various types of germ cells in a total number of 200 gametes.
(XLSX)
